# Contraceptive Decision-Making and the Importance of Side Effect Information Among a Sample of Latinas

**DOI:** 10.1089/whr.2021.0115

**Published:** 2022-01-31

**Authors:** Gaia Cicerchia, Lawrence D. Reid, Diana N. Carvajal

**Affiliations:** Department of Family and Community Medicine, University of Maryland School of Medicine, Baltimore, Maryland, USA.

**Keywords:** contraception, shared decision-making, vulnerable populations, quality of health care

## Abstract

***Context:*** U.S. Latinas have lower rates of contraceptive use and report more negative counseling experiences compared to non-Latina white women. Patient-centered approaches to contraceptive counseling are desired among Latinas and are also associated with patient satisfaction; yet, clinicians may not always use counseling methods that best support decision-making among marginalized groups.

***Objective:*** To examine Latinas' expectations of physician communication about contraceptive side effects, reported importance of personal knowledge about side effects, and the association of these with contraceptive use and use consistency.

***Materials and Methods:*** One hundred three self-identified Latinas aged 15–29 years at two urban federally qualified health centers completed a survey measuring factors associated with contraceptive use and consistency. Bivariate analysis was used to assess demographic and contraceptive information preferences. Associations between categorical variables were assessed using two-sided Fisher's exact tests. Continuous variables were compared using two-sided *t*-test.

***Results:*** The majority of respondents (85%) expected physicians to tell them about birth control side effects and reported that this information is important to use contraception, regardless of current contraceptive use. Among inconsistent and nonusers, importance of knowledge of side effects depended on expectations of information-sharing from physicians (*p* < 0.05).

***Conclusions:*** Expectations regarding side effect information-sharing by physicians and patient knowledge of side effects are important for the contraceptive decision-making process of this Latina group, regardless of contraceptive use. Clinicians engaging in contraceptive counseling should focus on providing clear and accurate information about side effects of discussed methods to facilitate informed decision-making and equitable, patient-centered contraceptive care for this vulnerable population.

## Introduction

Patient-centered contraceptive counseling can enhance contraceptive method satisfaction and use.^1^ However, access to and counseling about contraception is not equitable across racial and ethnic groups in the United States. Latinas have lower rates of contraceptive use when compared with non-Latina white women^2^ and are the least likely racial/ethnic group to use contraception at first sexual intercourse,^3^ despite evidence demonstrating a desire to use contraception.^4,5^ Patient-centered counseling is an important component of high-quality health care, which includes addressing a patient's specific preferences, desire for information, and participation in decision-making.^1^

Notably, patients desire more autonomy in contraceptive decision-making compared to other general health care decisions,^6^ and patient-centered approaches to contraceptive counseling have led to higher rates of contraceptive method satisfaction and use.^1^ Considering these trends, there is a need for more patient-centered contraceptive counseling for Latinas—beginning in adolescence and extending throughout the reproductive years.

A study of young, immigrant self-identified Latinas found that participants expressed clear regard for effective communication and trusting relationships with their primary care physicians when making decisions related to contraception.^4^ Yet, physicians who engage in contraceptive counseling may not communicate with their patients in a manner that best supports decision-making. Another study of young, low-income women in California found 50% of women reported choosing their method of contraception because of what their physician told them about that method.

However, patients still lacked basic information about the methods they were currently using.^7^ Compared to non-Latina white women, Latina and African-American women report more negative counseling experiences—as reflected by lower ratings of patient-centeredness, reports of coercion to use long-acting contraceptive methods, and incomplete information-sharing by their provider.^8–10^

Moreover, given the United States' history of reproductive coercion, including forced sterilization, committed against poor people and people of color, including Latinas,^11,12^ a better understanding of how counseling experiences impact contraceptive decision-making and use, is important to best support patients' contraceptive autonomy. As such, patient centered approaches for contraceptive counseling should be initiated early to support the spectrum of decision-making throughout the reproductive years.

One approach is shared decision-making (SDM)–which has proved to be acceptable for patients and specifically includes patient-centered information-sharing by the health care provider.^1,13^ SDM is a collaborative (between patients and clinicians) approach used for preference-sensitive decisions, in which multiple options may exist.^14^ This model encourages physicians to elicit patient preferences first and then tailor information-sharing (including information about potential side effects, alternative options, etc.) according to patient preferences to help facilitate their decisions.^15^

Given the need and evidence for an SDM approach, a better understanding of the role that physician information-sharing plays in the contraceptive decision-making of Latinas is important. Previous research has found that Latinas value acknowledgement of their contraceptive preferences. Specifically, concerns about side effects may play an important role in their use of contraception.^4,16–18^

However, it is not fully understood how Latinas perceive their physician's communication regarding potential contraceptive side effects or how their own knowledge about side effects impacts their contraceptive use.

The aim of this study is to examine the relationship between physician communication about contraceptive side effects, patient knowledge about contraceptive side effects, and contraceptive use and consistency. Specifically, this study explores patient expectations of physician information-sharing about side effects as well as the reported importance of knowledge about side effects for participants' contraceptive use and use consistency. This study aim is part of a larger study that sought to determine how patient-physician relationships and communication impact contraceptive decision-making among Latinas. A better understanding of the factors that impact contraceptive decision-making may help physicians best facilitate and support patient choices.

## Materials and Methods

### Participants, setting, and recruiting procedures

Self-identified Latinas aged 15–29 years were recruited from two primary care federally qualified health centers in a city in the Southeastern United States. Potential participants were approached in facility waiting rooms by a bilingual research assistant. Informed consent (>18 years old) or parental consent *and* assent (<18 years old) was obtained in the participant's language of choice (either English or Spanish). Each participant received a gift card as compensation.

Both facilities care for a predominantly low-income population of patients, many of whom are uninsured; a significant portion of the patient populations at each site are also of Latino/a/x descent. Participants included in the study were not pregnant or intending to become pregnant at the time of study consent. This human subjects research study was approved by the Institutional Review Board of the researchers' university.

### Data collection, instrument, and variables

A 50-item survey (with skip patterns) was administered to 103 participants. Question items were content-validated based on prior formative work with the population^16^ and, for this study, focused on participant expectations and values regarding communication with physicians specifically related to contraceptive side effects, contraceptive use and contraceptive use consistency.

Independent variables include the following: (A) Participant expectations with respect to physician communication about contraceptive side effects (“I expect my doctor will tell me about the side effects of birth control when we discuss my birth control options.”); and (B) Participant perceived importance of knowledge about contraceptive side effects as it relates to use (“Knowing the side effects of each of my birth control options is important for me to use it [birth control]”).

For both question items, response options ranged from *completely disagree* to *completely agree* (Likert scale 1–7). The responses were coded so that lower values corresponded to negative experiences and higher values corresponded to positive experiences.

Outcome variables included contraceptive use and consistent contraceptive use. *Contraceptive use* was defined as responding “Yes” to the question, “Are you (or your partner) currently using anything to prevent pregnancy now (contraception, birth control, family planning)?” If yes, participants were asked to choose from a list of contraceptives: “Which kind of contraception/birth control/family planning are you or your partner using now to keep from getting pregnant?.”^16^

Patients could report the use of more than one method. For patients who reported current use of contraception, determination of *use consistency* was modeled from previous research, determined by questions tailored to the type of contraceptive method the participant reported using.^19^ As an example, frequency of use as well as missing or late doses were assessed for shorter acting methods (oral contraceptives, contraceptive patch, contraceptive ring, and medroxyprogesterone injection).

Long-acting methods, including intrauterine devices and contraceptive implants were considered consistently used if participants reported using a device for at least 1 year and/or reported a plan to keep it for at least 1 year. Use consistency of other methods, including condoms, withdrawal, fertility awareness methods, and emergency contraception were also assessed. Withdrawal method and emergency contraception use were considered consistent if used during *every* sexual encounter over the previous 30 days; use of fertility awareness methods was considered consistent if used each month for the previous 60 days.

If participants reported more than one method, consistent use was defined as use of at least one method consistently. Responses were dichotomized to a binary variable for consistent contraception use of either “yes” or “no.”

### Statistical analysis

Bivariate analysis assessed demographic and contraceptive information preferences (expectations about physician communication of side effects and perceived importance of side effect knowledge) of consistent contraception users compared to inconsistent users and nonusers. Inconsistent contraception users were combined into a single group with nonusers who wanted to avoid pregnancy because these two groups seem likely to be similar in terms of their reasons for either not using contraception or using it inconsistently.

Both inconsistent users and nonusers included in this analysis reported wanting to avoid pregnancy. Contraceptive nonusers who did not want to avoid pregnancy or were ambivalent about pregnancy were excluded from analyses (*n* = 6). Associations between categorical variables were assessed using two-sided Fisher's exact tests; continuous variables were compared using two-sided *t*-test. The level of significance was set at α = 0.05. Data were analyzed using SAS 9.4 software (SAS Institute, Inc., Cary, NC).

## Results

Table 1 includes characteristics of the 97 participants in the study. Participants are predominantly from countries in Central America, Mexico, and the United States, although South American countries and some in the Caribbean are also represented. More than half (57%) of participants were consistently using at least one method of contraception at the time of data collection (*n* = 55). The remaining participants were either inconsistent contraceptive users (*n* = 10) or nonusers (who did not endorse wanting to be pregnant at the time of the survey, *n* = 32). The mean age of all participants was 21 years; however, consistent users were about 2 years older than inconsistent and nonusers, on average (22 years vs. 20 years; *p* < 0.05).

**Table 1.** Participant Characteristics by Consistent Contraceptive Use Status

Characteristic	Total (*n* = 97)	Contraception use	
		Consistent (*n* = 55)	Inconsistent or nonuser (*n* = 42)
Age			
15–21 years, %	53	44	64
22–29 years, %	47	56	36
Mean age, years (standard deviation)	21 (4)	22 (3)	20 (4)^*^
Country of origin, %			
El Salvador	25	25	24
Mexico	21	29	10
United States	21	18	24
Honduras	15	11	21
Other	19	16	21

*n*: number.

^*^ *p* < 0.05.

**FIG. 1.**
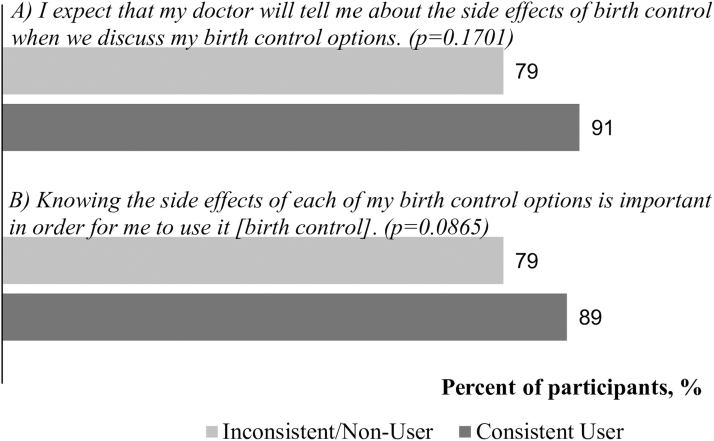
Percentage agreement with statements A and B by consistent contraception use status.

The majority of all participants (85%) agreed with the statement (A): “I expect my doctor will tell me about the side effects of birth control when we discuss my birth control options.” Similarly, 85% of all participants agreed with statement (B): “Knowing the side effects of each of my birth control options is important in order for me to use it [birth control].” Agreement with each statement did not differ statistically between consistent users and inconsistent and nonusers (*p* = 0.1701 and *p* = 0.0865, respectively; Fig. 1).

**FIG. 2.**
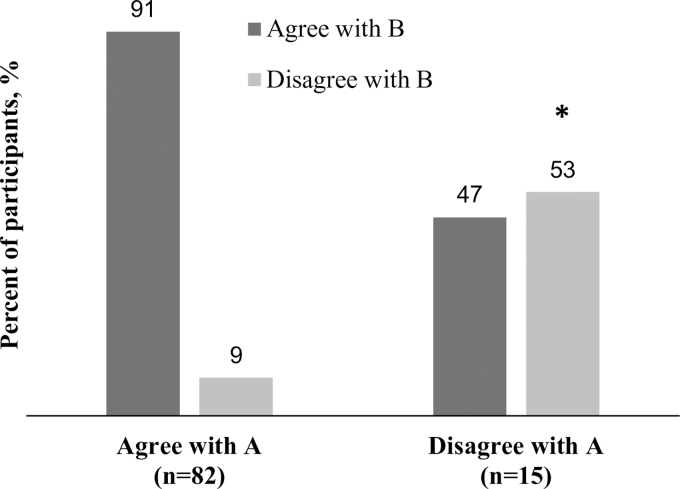
Distribution of responses to statements A and B. Statement A: I expect that my doctor will tell me about the side effects of birth control when we discuss my birth control options. Statement B: Knowing the side effects of each of my birth control options is important in order for me to use it (birth control). *p < 0.05.

**FIG. 3.**
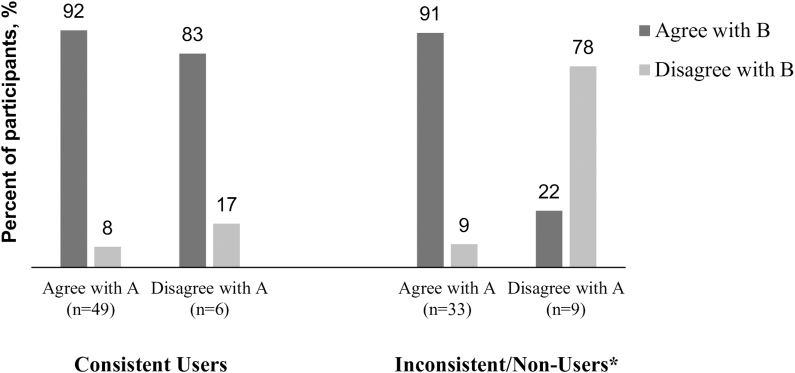
Distribution of responses to statements A and B by Contraceptive Use. Statement A: I expect that my doctor will tell me about the side effects of birth control when we discuss my birth control options. Statement B: Knowing the side effects of each of my birth control options is important in order for me to use it (birth control). *p < 0.05.

Nearly all participants who agreed with statement A also agreed with statement B (91%); more than half (53%) who disagreed with statement A also disagreed with statement B (*p* < 0.05; Fig. 2). Among consistent users, those who agreed with statement A also agreed with statement B (*p* = 0.4518). However, among nonusers/inconsistent users, agreement with statement B depended on agreement with statement A (*p* < 0.05; Fig. 3).

## Discussion

The results of this study indicate that among this group of Latinas, most participants expect their doctor to tell them about contraceptive side effects and agreed that knowing about side effects is important for their contraceptive use. This did not differ between consistent users and those who were nonusers/inconsistent users. Those who did not expect their doctor to inform them about side effects also tended to deny that knowing about side effects is important for deciding whether to use contraception. Consistent users agreed with both statements, while among inconsistent and nonusers, only those who agreed with statement A also tended to agree with statement B.

In summary, there were no differences between consistent users and others with respect to their report of their communication expectations from physicians or the importance of knowledge about side effects when making decisions to use contraception. While patient expectations about side effect information-sharing by the physicians and assertions of the importance of that knowledge for contraceptive use may be meaningfully linked, this study did not find statistical significance.

Yet, these results provide insight into the importance of the content of contraceptive counseling for these Latina participants within the clinical setting. Specifically, our data demonstrate that Latinas expect clear communication regarding contraceptive options and associated side effects regardless of whether they use contraception consistently, inconsistently, or not at all. Further, regardless of use status, this knowledge appears to be an important component of decision-making for the majority of participants.

This suggests that clear and accurate side-effect information during contraceptive counseling should be an integral component of patient-centered care for Latina patients, regardless of reported contraceptive use status. By doing so, physicians can meet patient expectations, equip patients with knowledge that is important for contraceptive use, and enhance patient autonomy within the decision-making process.

These findings support previous work, which has shown that the discussion of side effects during counseling does not deter or otherwise “negatively” impact patient use of contraception.^20^ It also adds to research regarding the factors that influence contraceptive decision-making among Latinas. Previous work has shown that concerns surrounding side effects are important for some Latinas; however, the role of patient-physician communication during counseling with respect to side effects remains unclear.^17,18^

These findings also build upon work about SDM and patient-centered care in contraceptive counseling, and specifically focuses on a sample of young, self-identified Latinas. Previous work among various populations has shown that SDM in contraceptive counseling increases satisfaction with counseling and with method choice.^1,13^

As such, it is not surprising that information-sharing regarding potential contraceptive side effects is an important component of the decision-making process among study participants. To our knowledge, however, the importance of expectations about side effect information-sharing from physicians, personal knowledge of side effects, and their relationship to use consistency has not been previously reported.

### Limitations

Limitations of this study include the relatively small sample size and lack of generalizability to other Latinas or other groups. This study was focused on a specific group of young, self-identified Latinas. Latina identity can encompass diverse populations who are both U.S. born and immigrants with ancestry from a variety of countries in Latin America, the Caribbean and Europe.

As such, our findings may not apply to Latinas outside of the study sample. Yet, this study is unique in that it assesses whether and how patient-physician communication regarding contraceptive side effects is important for patients. While previous studies have looked at larger sample sizes of participants, which have included Latinas, this study aimed to specifically understand the concerns of a Latina population that has been historically understudied.

### Implications for practice

Our study provides evidence that clear and accurate information-sharing about potential side effects of methods discussed is important regardless of current contraceptive use. In doing so, physicians can facilitate informed decision-making, autonomy, and just, equitable care for their patients. Such patient-centered counseling may help to mitigate contraceptive coercion among this vulnerable population with the ultimate aim of increasing patient satisfaction with counseling as well as with their chosen method.

## Conclusions

The United States has a long-standing history of imposing forced sterilization, unconsented contraceptive testing, and contraceptive coercion on women of color; Latinas are no exception.^11,21,22^ Moreover, concerns regarding equity and autonomy during contraceptive counseling persist as women of color still report being specifically discouraged from childbearing by providers,^21^ receiving incomplete contraceptive information from providers, and feeling implicit pressure to initiate certain contraceptive methods after provider-initiated counseling.^23^

Our study suggests patient-centered physician communication, specifically regarding desire and expectation for information about contraceptive side effects, may be an integral component of facilitating patient autonomy during contraceptive decision-making among Latinas. Future work should aim to understand whether these findings apply to a larger, more representative sample of Latinas and should include assessing the completeness of information-sharing regarding side effects during contraceptive counseling on the part of physicians. These study findings can act as a foundation with which to begin to provide more just, equitable, and patient-centered counseling to this historically marginalized population.

## Abbreviation Used

SDM: shared decision-making
